# Interactions between Food Additive Silica Nanoparticles and Food Matrices

**DOI:** 10.3389/fmicb.2017.01013

**Published:** 2017-06-07

**Authors:** Mi-Ran Go, Song-Hwa Bae, Hyeon-Jin Kim, Jin Yu, Soo-Jin Choi

**Affiliations:** Department of Applied Food System, Major of Food Science and Technology, Seoul Women's UniversitySeoul, South Korea

**Keywords:** silica, interaction, quantitative analysis, sugar, protein, lipid, mineral

## Abstract

Nanoparticles (NPs) have been widely utilized in the food industry as additives with their beneficial characteristics, such as improving sensory property and processing suitability, enhancing functional and nutritional values, and extending shelf-life of foods. Silica is used as an anti-caking agent to improve flow property of powered ingredients and as a carrier for flavors or active compounds in food. Along with the rapid development of nanotechnology, the sizes of silica fall into nanoscale, thereby raising concerns about the potential toxicity of nano-sized silica materials. There have been a number of studies carried out to investigate possible adverse effects of NPs on the gastrointestinal tract. The interactions between NPs and surrounding food matrices should be also taken into account since the interactions can affect their bioavailability, efficacy, and toxicity. In the present study, we investigated the interactions between food additive silica NPs and food matrices, such as saccharides, proteins, lipids, and minerals. Quantitative analysis was performed to determine food component-NP corona using HPLC, fluorescence quenching, GC-MS, and ICP-AES. The results demonstrate that zeta potential and hydrodynamic radius of silica NPs changed in the presence of all food matrices, but their solubility was not affected. However, quantitative analysis on the interactions revealed that a small portion of food matrices interacted with silica NPs and the interactions were highly dependent on the type of food component. Moreover, minor nutrients could also affect the interactions, as evidenced by higher NP interaction with honey rather than with a simple sugar mixture containing an equivalent amount of fructose, glucose, sucrose, and maltose. These findings provide fundamental information to extend our understanding about the interactions between silica NPs and food components and to predict the interaction effect on the safety aspects of food-grade NPs.

## Introduction

Nanotechnology and engineered nanoparticles (NPs) have attracted much attention for a wide range of applications in the field of chemical engineering, materials science, cosmetics, pharmaceutics, and medicine. NPs have been also widely applied to food products as additives, nutrient supplements, antimicrobial agents in food packaging, and delivery systems. According to Nanotechnology Consumer Products Inventory released in October 2013, the number of NPs-based commercial products in food and beverage have increased recently, of which gold, titanium dioxide, zinc oxide, and silica (SiO_2_) are among the most widely applied NPs (Vance et al., 2015). In particular, SiO_2_ NP is registered as a food additive E551 in the European Union (EU) and used as an anti-caking or anti-clumping agent (Dekkers et al., 2011; Wang et al., 2013). Humans are exposed to NPs through oral intake of NPs-containing food or beverage, which counts for 16% of total exposure (Vance et al., 2015). Oral ingestion being the third route of NP exposure, next to skin (58%) and inhalation (25%) (Vance et al., 2015; Cao et al., 2016), safety aspects of NPs to food sector duly need a critical consideration.

Toxicological effects of food-grade SiO_2_ NPs on biological systems have not been extensively explored compared to other commercial types. Moreover, most *in vitro* and *in vivo* studies have focused on biological responses upon exposure to SiO_2_ NPs, without considering potential interactions between NPs and biological or food components (Borak et al., 2012; Lee et al., 2014; Kim et al., 2016). As NPs in food are present in mixtures with food matrices containing carbohydrates, proteins, lipids, minerals, and other trace elements, interactions between NPs and food components can be critical factors affecting potential toxicity, oral absorption, biodistribution, and efficacy of NPs. It was reported that NPs affect nutrient absorption (Mahler et al., 2012; Dorier et al., 2015), but the interactions between food-grade NPs and food components have not been well explored. On the other hand, food components could also influence the absorption and toxicity of NPs as well (Wang et al., 2014; Bohmert et al., 2015; Docter et al., 2015; Lichtenstein et al., 2015; Jiang et al., 2016). Indeed, our previous research suggested that oral absorption of food-grade SiO_2_ NPs is highly affected by food components, showing 2.4- and 2.5-fold enhanced absorption efficiencies in the presence of albumin and glucose, respectively (Lee et al., 2017).

The aim of the present study was to determine the interactions between SiO_2_ NPs and food matrices. NP interactions with honey, skim milk, olive oil, or phosphate buffered saline (PBS), which represent saccharide, protein, lipid, and mineral matrices, respectively, were analyzed quantitatively. In addition, the role of trace nutrients on the NP interaction with saccharides and casein (major components in honey and skim milk, respectively) was also investigated.

## Materials and methods

### Materials and characterization

Food-grade amorphous SiO_2_ NPs were purchased from Evonik Industries AG (Essen, Germany). Materials used were as follow: acacia honey (Dongsuh Food Co., Ltd., Seoul, Republic of Korea), D-(+)-glucose (Sigma-Aldrich, St. Louis, MO, USA), D-(−)-fructose (Sigma-Aldrich), sucrose (Sigma-Aldrich), D-(+)-maltose monohydrate (Sigma-Aldrich), skim milk powder (Seoul Milk, Seoul, Republic of Korea), casein sodium salt from bovine milk (Sigma-Aldrich), extra virgin olive oil (imported from Spain, Beksul, CJ CheilJedang, Seoul, Republic of Korea), 37-component fatty acid methyl esters (FAMEs; Sigma-Aldrich), hexane (Sigma-Aldrich), sodium hydroxide (Sigma-Aldrich), biphenyl (Sigma-Aldrich), methanol (Samchun Chemical Co., Ltd., Gyeonggi-do, Republic of Korea), acetonitrile (HPLC grade, Samchun Chemical Co., Ltd.), water (HPLC grade, Samchun Chemical Co., Ltd.), and PBS buffer (NaCl 137 mM, KCl 2.7 mM, Na_2_HPO_4_ 10 mM, KH_2_PO_4_ 1.8 mM; Dongin Biotech. Co., Republic of Korea).

Particle size and morphology were determined by scanning electron microscopy (SEM; FEIQUANTA 250 FEG, Hillsboro, OR, USA). Zeta potentials and hydrodynamic radii of NPs in aqueous suspension and food matrices were measured with Zetasizer Nano System (Malvern Instruments, Worcestershire, UK). Stock solution of SiO_2_ NPs [50 mg/ml in distilled water (DW)] was prepared, stirred for 30 min, and diluted to designated concentrations just prior to experiments.

### Solubility

Particles (5 mg/ml) were dispersed in 10% honey, 1 mg/ml of skim milk solution, olive oil, and PBS buffer, respectively. After different incubation times at 25°C, supernatants were collected by ultracentrifugation (16,000 × g) for 15 min, and analysis of Si in the supernatants was performed as described previously (Paek et al., 2014).

### Interaction between NPs and saccharides

Different concentrations (1, 2, 5, and 10%) of acacia honey were prepared in DW and incubated with 5 mg/ml NPs with shaking at different temperatures (4, 25, and 40°C). 10% concentration of honey, containing ~42.4% fructose, ~29.6% glucose, ~0.2% sucrose, and ~0.1% maltose, was used as the highest concentration due to high viscosity over this level. Fructose, glucose, sucrose, and maltose at each concentration of 1, 2, and 5% were mixed and used as sugar mixtures to mimic honey matrix containing only equivalent amounts of each sugar, without trace nutrients. After designated incubation times (1, 24, 48 h, and 7 d), the samples were centrifuged at 23,000 × g for 1 h. The supernatants were analyzed after washing with distilled and deionized water (DDW) or without washing, and then, filtered through syringe filter (Agela Technologies, Wilmington, DE, USA). Saccharide concentrations were quantified by high performance liquid chromatography (HPLC) using a Shimadzu HPLC system (Kyoto, Japan), equipped with RID-10A refractive index detector, on a Hypersil APS-2 column (250 × 4.6 mm i.d., 5 μm, 120 Å, Thermo Fisher Scientific, MA, USA). The mobile phase was acetonitrile:water (80:20, v/v) and flow rate was set at 1 ml/min. Column temperature was maintained at a constant 40°C and injection volume of sample was 20 μl. Each experiment was repeated three times on separate days.

### Interaction between NPs and proteins

Different concentrations of SiO_2_ NPs (1, 2.5, and 5 mg/ml) were suspended in 1 mg/ml of skim milk solution (in DW) or 0.35 mg/ml of casein solution (in DW), and incubated with shaking at different temperatures (4, 25, and 40°C). After designated incubation times (1, 24, 48 h, and 7 d), the suspensions were subjected to protein fluorescence quenching analysis using a luminescence spectrometer (SpectraMax® M3, Molecular Devices, CA, USA). Excitation wavelength was set at 280 nm and fluorescence emission intensity was measured at wavelength from 300 to 420 nm. Quenching ratio was calculated as (I0-I)/I0, where I0 and I stand for basal fluorescence emission intensity in controls (NPs-untreated proteins) and experimental groups (NPs-treated proteins), respectively. Each experiment was repeated three times on separate days.

### Interaction between NPs and lipids

SiO_2_ NPs (50 mg/ml) were dispersed in olive oil for 30 min and diluted to 20 mg/ml prior to experiments. NPs suspended in olive oil were incubated with shaking for 1, 24, and 48 h at different temperatures (4, 25, and 40°C). After centrifugation at 23,000 × g for 1 h, fatty acid methyl esters were prepared by alkaline transmethylation (Liang et al., 2011). The supernatants (1 μl) were spiked with internal standard solution (final concentration of 100 μg/ml), and 1 ml of 0.4 M NaOH-CH_3_OH was added and reacted for 5 min with ultrasonification. The methyl esters were extracted with 5 ml of hexane, diluted to 3-folds, and analyzed by gas chromatography-mass spectrometry (GC-MS). A standard mixture (FAMEs, 1 ml of 10 mg/ml) was dissolved in 9 ml of hexane and the standard solution was diluted to 10, 20, 50, 100, 200, and 500 μg/ml concentrations, and then, spiked with internal standard biphenyl (100 μg/ml). The adsorbed fatty acids on NPs were calculated after subtraction of reduced fatty acids in the supernatants from those in olive oil control. Each experiment was repeated three times on separate days. All GC-MS analyses were performed with an Agilent 5977E GC-MSD system (Agilent Technologies, CA, USA), including 7820A GC instrument coupled with a 5977E MS detector. A StabilWax® capillary column (30 m × 0.25 mm, 0.25 μm thickness, Restek, PA, USA) was used, and column temperature was hold for 1 min at 50°C, programmed from 50 to 200°C at the rate of 25°C/min, from 200 to 230°C at the rate of 3°C/min, and then hold for 23 min at 230°C. The injection temperature was kept at 250°C and the carrier gas was helium. The column flow was 1 ml/min and a sample of 3 μl was injected with a split ratio of 5:1. The ion source temperature was 230°C and the samples were ionized by electron impact ionization at 70 eV. Selected-ion monitoring was performed at m/z 55, 67, 74, 79, and 87.

### Interaction between NPs and minerals

Two different concentrations (5 and 10 mg/ml) of SiO_2_ NPs were dispersed in 1 ml PBS and reacted with shaking for various times (1, 24, 48 h, and 7 d) at different temperatures (4, 25, and 40°C). To remove unbound minerals from NPs, the samples were centrifuged at 23,000 × g for 1 h and washed with DDW. This procedure was repeated three times. The aliquots were digested with 10 ml of ultrapure nitric acid at ~180°C and diluted with 3 ml of DDW. After filtering through syringe filter (0.22 μm, Agela Technolgies), total Na concentrations were determined by inductively coupled plasma-atomic emission spectroscopy (ICP-AES, JY2000 Ultrace, HORIBA Jobin Yvon, longjumeau, France). Each experiment was repeated three times on separate days.

### Statistical analysis

Results were expressed as means ± standard deviations. Experimental values were compared with corresponding untreated control values. Statistical analysis was performed using the Student's test for unpaired data and one-way analysis of variance (ANOVA) with Tukey's Test in SAS Version 9.4 (SAS Institute Inc., Cary, NC, USA) was carried out to determine the significances of intergroup differences. Statistical significance was accepted for *p*-values of <0.05.

## Results

### Characterization

**Figure 1:**
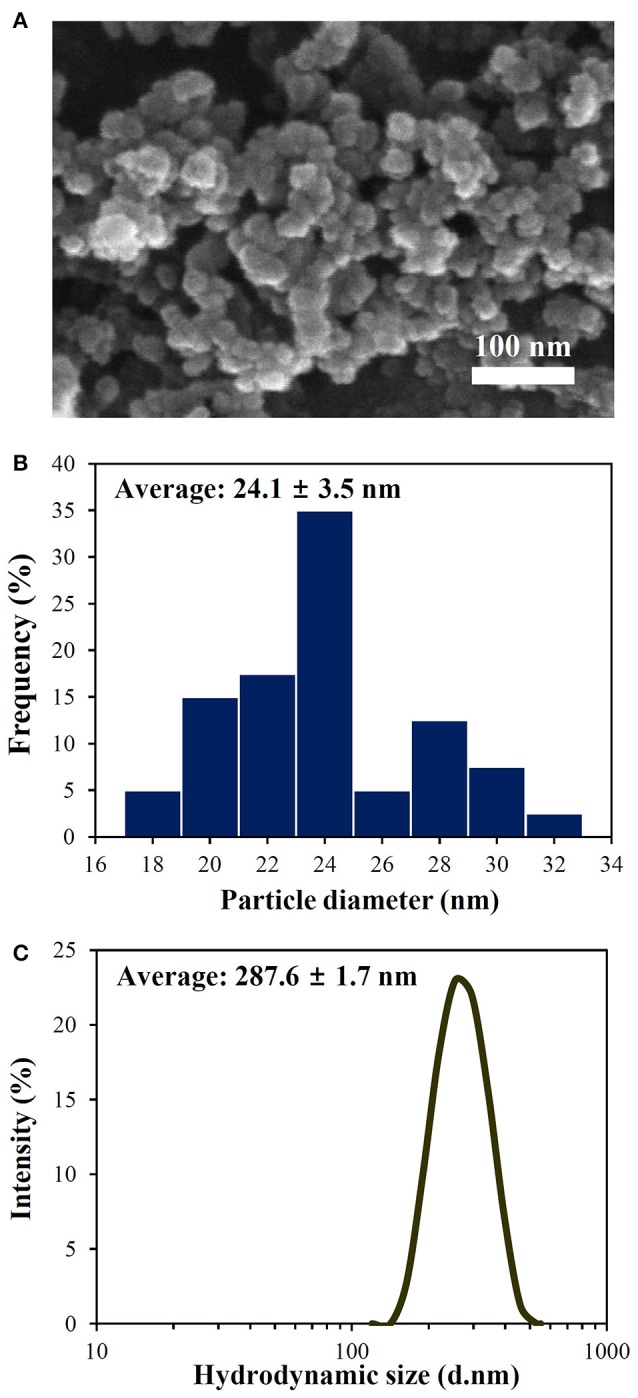
**(A)** SEM image, **(B)** size distribution of primary particles, and **(C)** hydrodynamic diameters of SiO_2_ NPs in DW. Primary particle size distribution was measured by randomly selecting 200 particles from the SEM image.

Particle morphology, primary particle size, and size distribution were examined by SEM. Figure 1 shows that food-grade SiO_2_ NPs dispersed in DW had irregular particle morphology with an average particle size of 24.1 ± 3.5 nm. Zeta potential and hydrodynamic size of SiO_2_ NPs were determined to be −28.2 ± 1.0 mV and 287.6 ± 1.7 nm, respectively. Solubility of SiO_2_ NPs in DW was not detected and did not increase in the presence of food matrices.

### Interaction between NPs and saccharides

Zeta potential values of SiO_2_ NPs in honey changed to less negative charges as honey concentration and incubation time increased (Table 1), regardless of temperature (Table 2). Hydrodynamic radii of SiO_2_ NPs in honey also significantly increased as honey concentration increased (Table 1), without effect of incubation time and temperature (Tables 1, 2). However, zeta potentials and hydrodynamic radii of NPs could not be detected at concentrations of more than 2% honey after incubation for 7 d due to the formation of high aggregation (Supplementary Figure 1).

**Table 1:** Zeta potentials and hydrodynamic diameters of SiO_2_ NPs in honey at 25°C.

**Zeta potential (mV)**						**Hydrodynamic size (nm)**					
**DW**	**Time**	**Concentration (%)**				**DW**	**Time**	**Concentration (%)**			
		**1%**	**2%**	**5%**	**10%**			**1%**	**2%**	**5%**	**10%**
−28.2 ± 1.0^A,a^	1 min	−27.8 ± 0.8^A,a^	−28.4 ± 0.4^A,a^	−25.0 ± 0.9^B,b^	−20.6 ± 1.0^BC,c^	287.6 ± 1.7^A,a^	1 min	311.5 ± 39.2^A,a^	346.1 ± 52.7^B,a^	537.4 ± 65.2^B,b^	871.0 ± 36.1^B,c^
	1 h	−27.0 ± 0.9^AB,a^	−24.4 ± 0.5^B,b^	−25.0 ± 0.5^B,b^	−21.6 ± 0.4^B,c^		1 h	310.9 ± 4.5^A,a^	355.2 ± 7.3^B,a^	533.7 ± 75.4^B,b^	882.7 ± 18.4^B,c^
	24 h	−26.7 ± 0.4^AB,ab^	−25.3 ± 0.8^B,b^	−22.7 ± 0.9^C,c^	−19.1 ± 0.8^C,d^		24 h	302.2 ± 14.0^A,a^	367.1 ± 0.7^B,a^	533.2 ± 29.2^B,b^	840.5 ± 111.3^B,c^
	48 h	−25.6 ± 1.0^B,b^	−24.9 ± 1.0^B,b^	−22.1 ± 0.4^C,c^	−18.7 ± 0.6^C,d^		48 h	298.9 ± 9.7^A,a^	382.9 ± 15.7^B,a^	565.4 ± 43.5^B,b^	799.5 ± 137.2^B,c^
	7 d	−19.1 ± 0.8^C^	ND	ND	ND		7 d	304.5 ± 17.5^A^	ND	ND	ND

*Different letters (A–C) in the same column indicate significant differences between incubation times (p < 0.05). Different letters (a–d) in the same row indicate significant differences between honey concentrations (p < 0.05). Zeta potential and hydrodynamic radius of NPs in DW were measured in the absence of honey as a control. Zeta potentials and hydrodynamic diameters could not be detected at 2–5% after incubation for 7 d due to aggregation (See Supplementary Figure 1). ND, not determined*.

**Table 2:** Zeta potentials and hydrodynamic diameters of SiO_2_ NPs in honey, sugar mixtures, skim milk, casein, and PBS buffer after incubation for 24 h at different temperatures.

**Matrix**	**Zeta potential (mV)**			**Hydrodynamic size (nm)**		
	**Temperature (°C)**			**Temperature (°C)**		
	**4°C**	**25°C**	**40°C**	**4°C**	**25°C**	**40°C**
Honey (10%)	−18.7 ± 2.1	−19.1 ± 0.8	−17.2 ± 0.7	823.9 ± 13.8	840.5 ± 111.3	846.1 ± 126.2
Sugar mixture (5%)	−27.8 ± 1.6	−26.5 ± 0.4	−27.0 ± 0.6	272.7 ± 10.5	271.8 ± 0.4	273.0 ± 18.8
Skim milk (1 mg/ml)	−25.5 ± 1.8	−25.6 ± 1.3	−25.3 ± 1.3	259.6 ± 6.8	261.7 ± 1.6	268.0 ± 0.5
Casein (0.35 mg/ml)	−34.8 ± 0.6	−33.7 ± 0.7	−35.1 ± 1.8	299.9 ± 7.0	290.3 ± 8.2	290.4 ± 8.8
PBS	−36.4 ± 0.4	−36.7 ± 1.0	−35.5 ± 0.9	251.0 ± 4.2	249.6 ± 4.3	251.7 ± 1.7

*No significant differences between different temperatures were found (p > 0.05). Sugar mixtures consist of equivalent amounts of fructose, glucose, sucrose, and maltose. Concentration of casein solution was adjusted to have an equivalent amount of protein content in 1 mg/ml of skim milk solution*.

Since honey contains different amounts of saccharides and other trace nutrients, the interactions between SiO_2_ NPs and saccharides were further evaluated using sugar mixtures composed of equivalent amounts of fructose, glucose, sucrose, and maltose, in order to investigate the effects of only saccharides on interactions. It is worth noting that these are major four saccharide components in acacia honey. The highest concentration of each saccharide was set at 5%, based on maximum solubility of both fructose and maltose in DW. Zeta potentials of SiO_2_ NPs in sugar mixtures did not significantly change, except at 5%, and were not affected by incubation time and temperature (Tables 2, 3). Interestingly, hydrodynamic sizes of NPs decreased in the presence of sugar mixtures in a time-dependent manner (Table 3). No aggregation tendency was observed in sugar mixtures (Supplementary Figure 1).

**Table 3:** Zeta potentials and hydrodynamic diameters of SiO_2_ NPs in sugar mixtures at 25°C.

**Zeta potential (mV)**					**Hydrodynamic size (nm)**				
**DW**	**Time**	**Concentration (%)**			**DW**	**Time**	**Concentration (%)**		
		**1%**	**2%**	**5%**			**1%**	**2%**	**5%**
−28.2 ± 1.0^A,a^	1 min	−28.6 ± 0.1^A,a^	−27.8 ± 0.7^A,ab^	−26.2 ± 0.2^A,b^	287.6 ± 1.7^A,a^	1 min	270.4 ± 3.7^BC,b^	285.2 ± 8.7^A,a^	288.7 ± 6.4^A,a^
	1 h	−27.8 ± 1.2^A,a^	−27.6 ± 0.6^A,a^	−26.7 ± 0.6^A,a^		1 h	275.6 ± 3.9^B,b^	285.6 ± 5.2^A,a^	289.7 ± 2.1^A,a^
	24 h	−27.1 ± 0.5^A,a^	−27.3 ± 0.5^A,ab^	−26.5 ± 0.4^A,b^		24 h	264.0 ± 4.3^C,b^	284.6 ± 7.4^AB,a^	271.8 ± 0.4^B,b^
	48 h	−28.6 ± 1.0^A,a^	−27.9 ± 1.1^A,a^	−26.6 ± 2.0^A,a^		48 h	263.9 ± 3.3^C,b^	270.4 ± 1.8^AB,b^	270.7 ± 5.9^B,b^
	7 d	−28.1 ± 0.8^A,a^	−27.9 ± 0.7^A,a^	−26.2 ± 0.4^A,b^		7 d	262.3 ± 2.0^C,b^	252.0 ± 18.6^B,b^	257.8 ± 8.6^B,b^

*Different letters (A–C) in the same column indicate significant differences between incubation times (p < 0.05). Different letters (a,b) in the same row indicate significant differences between sugar mixture concentrations (p < 0.05). Sugar mixtures consist of equivalent amounts of fructose, glucose, sucrose, and maltose. Zeta potential and hydrodynamic radius of NPs in DW were measured in the absence of sugar mixtures as a control*.

**Figure 2:**
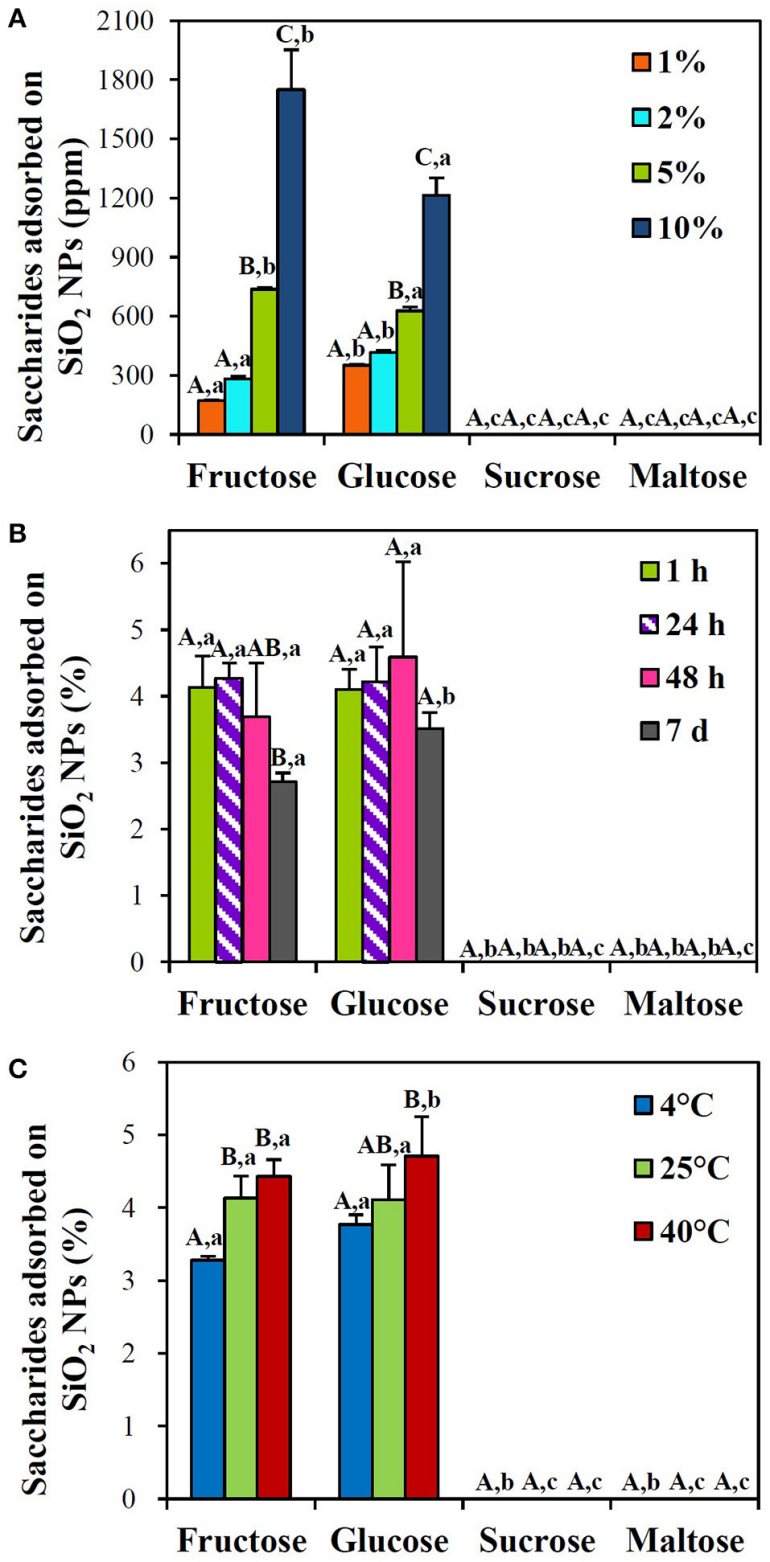
HPLC analysis of the interactions between SiO_2_ NPs and saccharides in acacia honey with respect to **(A)** honey concentrations after incubation for 1 h at 25°C, **(B)** incubation times at 10% honey at 25°C, and **(C)** temperatures after 1 h at 10% honey. Different letters in majuscule (A–C) indicate significant differences **(A)** between honey concentrations, **(B)** between incubation times, and **(C)** between temperatures, respectively (*p* < 0.05). Different letters in minuscule (a–c) indicate significant differences between saccharide types (*p* < 0.05).

When the interactions between SiO_2_ NPs and saccharides in honey were quantified by HPLC, Figure 2A shows that fructose and glucose, the most abundant two saccharides in honey, interacted with SiO_2_ NPs in a concentration-dependent manner at 25°C, while no interactions between SiO_2_ NPs and sucrose or maltose were found. NP interaction with fructose was slightly higher than that with glucose at more than 5% honey (*p* < 0.05). However, the interactions between SiO_2_ NPs and glucose or fructose in honey were not affected by incubation time (Figure 2B) for 48 h. Meanwhile, the interactions between SiO_2_ NPs and glucose or fructose increased as temperature increased, especially, low interaction levels were detected at 4°C, compared to 25 and 40°C (Figure 2C).

**Figure 3:**
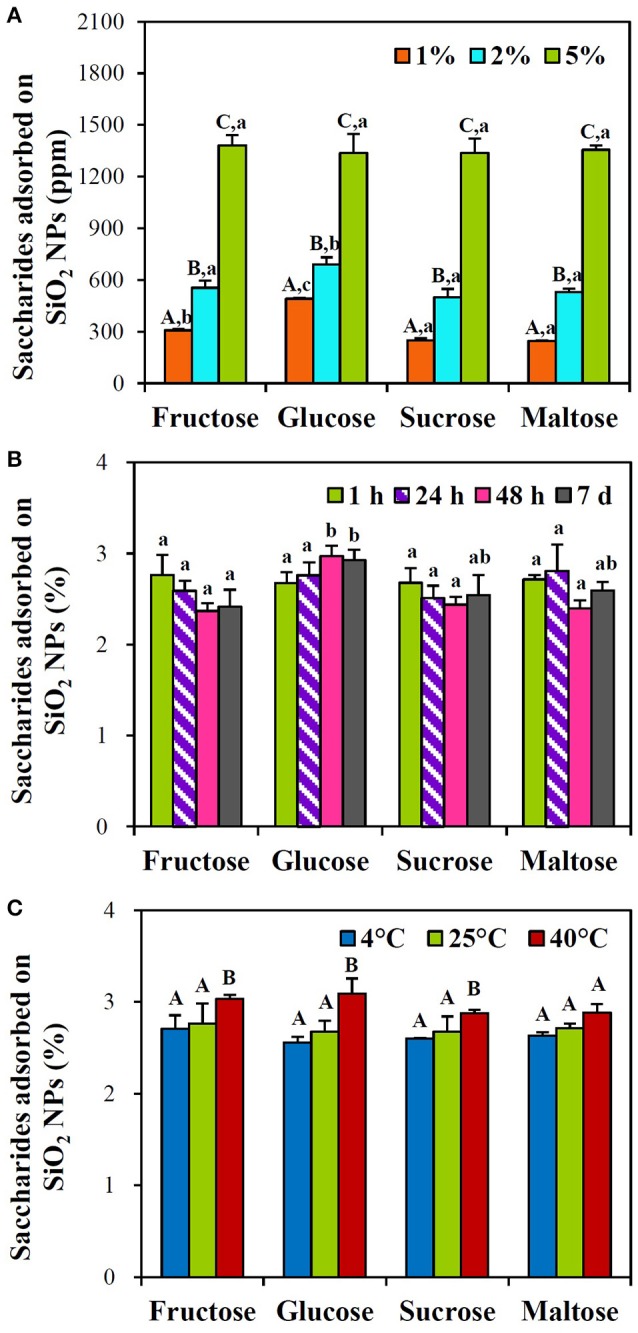
HPLC analysis of the interactions between SiO_2_ NPs and saccharides in sugar mixtures with respect to **(A)** sugar concentrations after incubation for 1 h at 25°C, **(B)** incubation times at 5% sugar mixture at 25°C, and **(C)** temperatures after 1 h at 5% sugar mixture. Sugar mixtures contain equivalent amounts of fructose, glucose, sucrose, and maltose. Different letters in majuscule (A–C) indicate significant differences **(A)** between sugar mixture concentrations and **(C)** between temperatures, respectively (*p* < 0.05). Different letters in minuscule (a–c) indicate significant differences between saccharide types (*p* < 0.05). No statistical differences **(B)** between incubation times and **(C)** between saccharide types were found (*p* > 0.05).

Similar tendency was found in sugar mixtures containing equivalent amounts of fructose, glucose, sucrose, and maltose. Concentration-, but not time-dependent interactions between SiO_2_ NPs and saccharides were found (Figures 3A,B), and these interactions significantly increased when incubation temperature increased at 40°C (Figure 3C). In particular, all different types of saccharide interacted with NPs in a similar manner. Significant differences in the interactions between saccharide types were not remarkably found (*p* > 0.05). Overall, < 4.6 and 3.1% of saccharides in honey and sugar mixtures, respectively, were determined to interact with SiO_2_ NPs. It should be noted that quantitative analysis was performed after ultracentrifugation of reacted NPs with saccharides, collecting precipitated NPs, and detaching adsorbed saccharides on NPs with HPLC eluent. Whereas, no adsorbed saccharide was detected after washing the centrifuged and precipitated NPs with DDW.

### Interaction between NPs and proteins

Skim milk and its main protein component, casein were used to determine the interactions between SiO_2_ NPs and proteins. Skim milk solution at 1 mg/ml (in DW) was applied for protein fluorescence measurement, because this concentration exhibited the highest fluorescence intensity without precipitation (data not shown). Based on casein content in skim milk, 0.35 mg/ml of casein solution was used for comparative study. Zeta potential values of SiO_2_ NPs significantly changed to less negative direction in skim milk solution, whereas more negative zeta potentials were found in casein solution (Table 4). Effect of temperature on the interactions was not found (Table 2; *p* > 0.05). Aggregation was observed after incubation for 7 d at 25 and 40°C in both skim milk and casein solutions treated with NPs (Supplementary Figure 1). Meanwhile, hydrodynamic radii of NPs were not remarkably influenced by incubation time, temperature, and the presence of skim milk or casein (Tables 2, 4).

**Table 4:** Zeta potentials and hydrodynamic diameters SiO_2_ NPs in skim milk and casein solutions at 25°C.

**Zeta potential (mV)**				**Hydrodynamic size (nm)**			
**DW**	**Time**	**Skim milk (1 mg/ml)**	**Casein (0.35 mg/ml)**	**DW**	**Time**	**Skim milk (1 mg/ml)**	**Casein (0.35 mg/ml)**
−28.2 ± 1.0^A,a^	1 min	−27.3 ± 0.8^AB,a^	−43.9 ± 3.6^C,b^	287.6 ± 1.7^A,a^	1 min	284.5 ± 4.7^A,a^	317.87 ± 1.42^B,b^
	1 h	−27.1 ± 0.8^AB,a^	−36.0 ± 1.4^B,b^		1 h	322.0 ± 11.6^C,b^	344.3 ± 18.5^C,b^
	24 h	−25.6 ± 1.3*A*^B,a^	−33.7 ± 0.7^B,b^		24 h	268.0 ± 9.2^B,b^	292.0 ± 4.3^A,a^
	48 h	−25.4 ± 1.2^B,b^	−32.8 ± 2.4^AB,c^		48 h	270.6 ± 4.8^B,b^	274.2 ± 1.8^A,b^
	7 d	−24.0 ± 1.9^C,b^	−32.4 ± 1.6^A,c^		7 d	296.6 ± 0.7^A,b^	287.9 ± 1.6^A,a^

*Different letters (A–C) in the same column indicate significant differences between incubation times (p < 0.05). Different letters (a–c) in the same row indicate significant differences between skim milk and casein solutions (p < 0.05). Concentration of casein solution was adjusted to have an equivalent amount of protein content in 1 mg/ml of skim milk solution. Zeta potential and hydrodynamic radius of NPs in DW were measured in the absence of skim milk and casein solutions as a control*.

**Figure 4:**
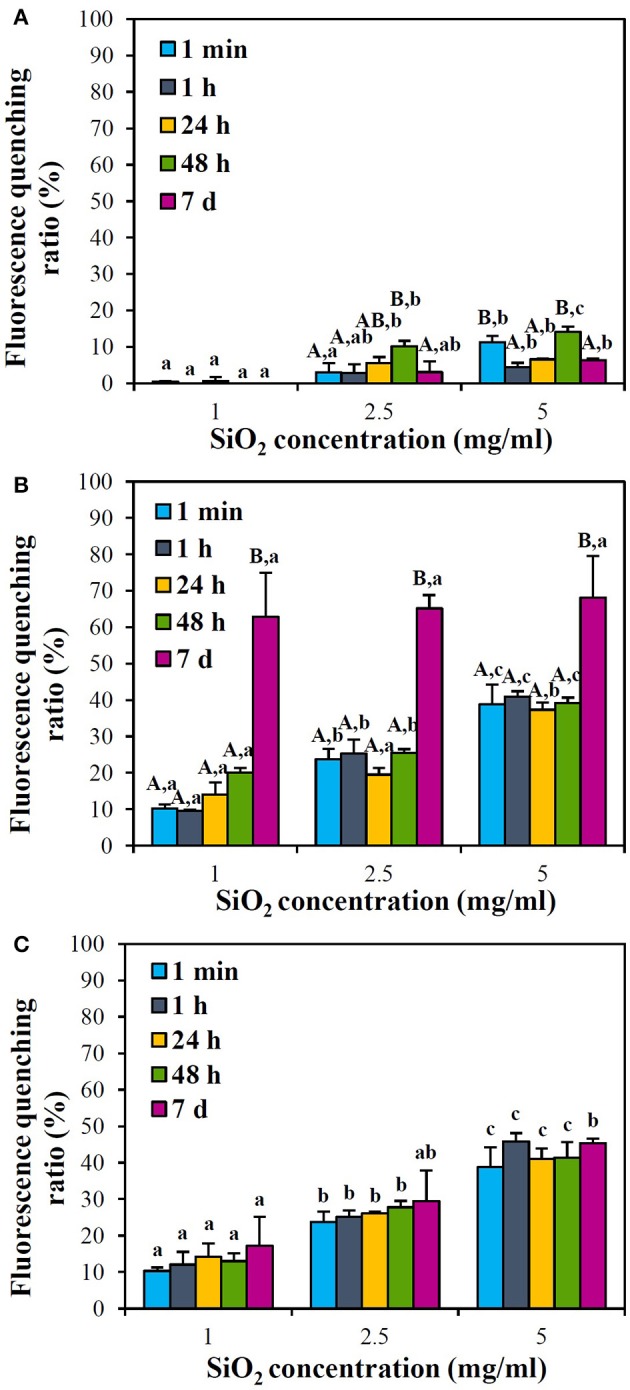
Fluorescence quenching ratios of skim milk solution (1 mg/ml) in the presence of SiO_2_ NPs with respect to NP concentrations at **(A)** 4°C, **(B)** 25°C, and **(C)** 40°C. Different letters in majuscule (A,B) and in minuscule (a–c) indicate significant differences between incubation times and between NP concentrations, respectively (*p* < 0.05). **(C)** No statistical differences between incubation times were found (*p* > 0.05).

Interactions between SiO_2_ NPs and protein were further estimated by measuring protein fluorescence quenching ratio in the presence of NPs. Figure 4 shows that fluorescence quenching ratio immediately increased just after adding NPs in skim milk solution at all temperatures tested in a NP concentration-dependent manner. Remarkably high fluorescence quenching ratio (more than 60% fluorescence quenching) was induced at 25°C after incubation for 7 d (Figure 4B), where aggregation was observed (Supplementary Figure 1). Meanwhile, fluorescence quenching ratio significantly increased at 25 and 40°C compared to 4°C. However, overall fluorescence quenching ratios were < 40% and blue or red shift was not observed.

**Figure 5:**
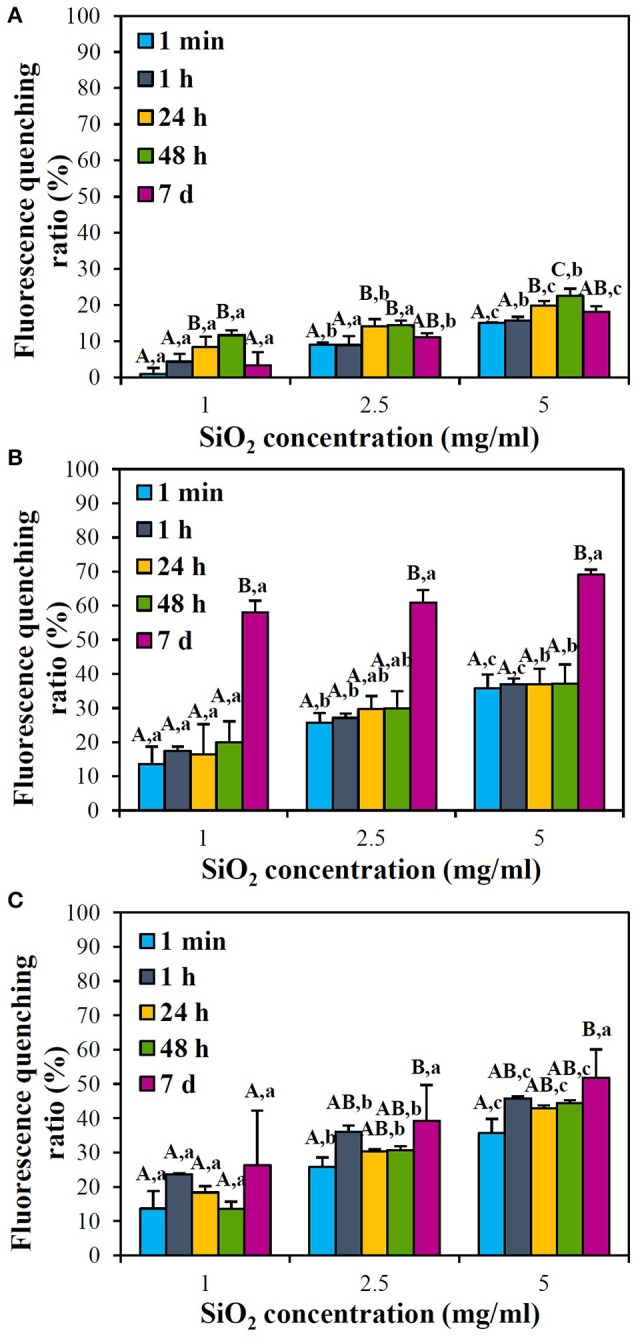
Fluorescence quenching ratios of casein solution (0.35 mg/ml) in the presence of SiO_2_ NPs with respect to NP concentrations at **(A)** 4°C, **(B)** 25°C, and **(C)** 40°C. Different letters in majuscule (A–C) and in minuscule (a–c) indicate significant differences between incubation times and between NP concentrations, respectively (*p* < 0.05).

When the interactions between SiO_2_ NPs and casein, a major protein in skim milk, was assessed, similar tendency was obtained (Figure 5). NP concentration-dependent increase in fluorescence quenching ratio was found, and 60–70% quenching was induced by NPs at 25°C after 7 d of incubation. Interactions between NPs and casein at 25°C were comparable to those at 40°C, except at 7 d post-incubation, while significantly reduced fluorescence quenching was observed at 4°C. Blue shift was only observed at 40°C after 48 h of incubation (Supplementary Figure 2). On the other hand, fluorescence quenching ratios of NPs in 0.35 mg/ml of casein solution (Figure 5) were similar to those in 1 mg/ml of skim milk solution (Figure 4).

### Interaction between NPs and lipids

**Figure 6:**
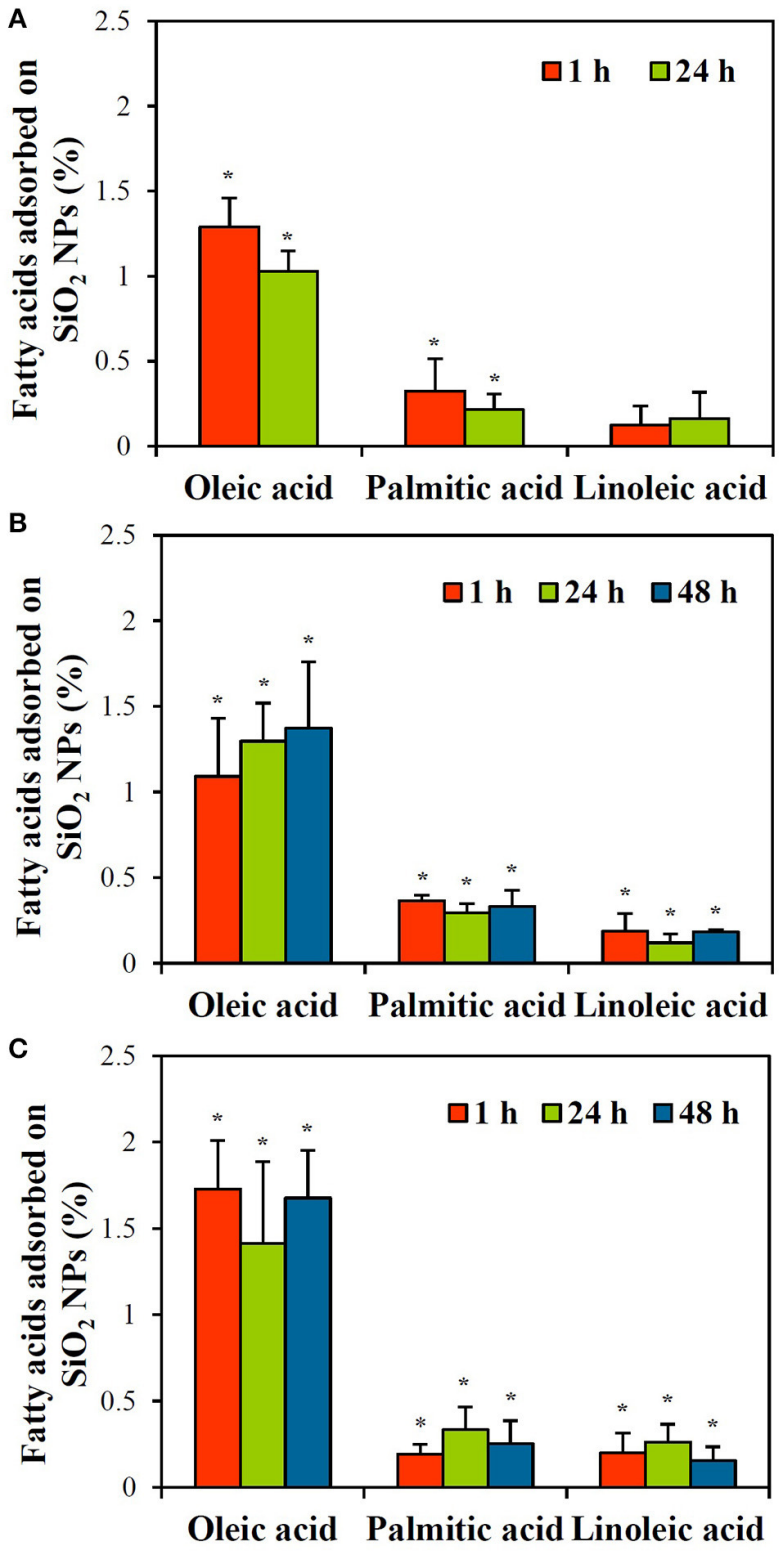
GC-MS analysis of the interactions between SiO_2_ NPs and fatty acids in olive oil with respect to incubation times at **(A)** 4°C, **(B)** 25°C, and **(C)** 40°C. ^*^Denotes significant difference from olive oil control (*p* < 0.05). No statistical differences were found between different incubation times (*p* > 0.05). Interactions at 4°C after 48 h could not be detected due to NP gelation in olive oil (See Supplementary Figure 1).

Zeta potentials and hydrodynamic radii of SiO_2_ NPs in the presence of olive oil could not be detected because of intense yellow color and high viscosity of olive oil. GC-MS analysis reveals that olive oil used in the presence study contains ~59.3% oleic acid, ~11.4% palmitic acid, and ~5.7% linoleic acid. Because SiO_2_ NPs have hydrophilic surface characteristics (Jesionowski and Krysztafkiewicz, 2002) and slightly bound fatty acids on the surface of NPs are likely to be easily detached during washing with organic solvents, the interactions between SiO_2_ NPs and lipids were estimated by measuring fatty acid composition in the supernatant after reaction with NPs, followed by subtraction of reduced fatty acids from those in olive oil control. The results show that < 1.8% oleic acid and 0.4% palmitic and linoleic acids interacted with SiO_2_ NPs, and the effects of incubation time and temperature on the interactions were not found (Figure 6; *p* > 0.05). However, interactions at 4°C after 48 h and at all temperatures tested after 7 d could not be detected, resulted from gelation of SiO_2_ NPs in olive oil (Supplementary Figure 1).

### Interaction between NPs and minerals

PBS buffer was used to investigate the interaction effects of minerals on the surface of SiO_2_ NPs. Zeta potentials of NPs significantly and rapidly changed to more negative charge in PBS without effects of incubation time, NP concentration, and temperature (Tables 2, 5). DLS data reveal that significantly reduced size distribution of NPs was found just after adding NPs in PBS and their reduced hydrodynamic radii were maintained for 7 d. Remarkable aggregation was not observed even after incubation for 7 d (Supplementary Figure 1).

**Table 5:** Zeta potentials and hydrodynamic diameters SiO_2_ NPs in PBS buffer at 25°C.

**Zeta potential (mV)**				**Hydrodynamic size (nm)**			
**DW**	**Time**	**SiO**_**2**_ **concentration**		**DW**	**Time**	**SiO**_**2**_ **concentration**	
		**5 mg/ml**	**10 mg/ml**			**5 mg/ml**	**10 mg/ml**
−28.2 ± 1.0^A,a^	1 min	−36.3 ± 1.0^B,b^	−35.8 ± 0.4^B,b^	287.6 ± 1.7^A,a^	1 min	269.4 ± 5.5^B,b^	269.7 ± 4.7^B,b^
	1 h	−37.3 ± 1.2^B,b^	−36.5 ± 1.0^B,b^		1 h	250.4 ± 2.1^C,b^	250.0 ± 4.7^C,b^
	24 h	−36.4 ± 1.8^B,b^	−36.7 ± 1.0^B,b^		24 h	247.9 ± 1.7^C,b^	249.6 ± 4.3^C,b^
	48 h	−36.2 ± 1.1^B,b^	−36.3 ± 0.5^B,b^		48 h	247.4 ± 2.3^C,b^	246.7 ± 1.4^C,b^
	7 d	−37.1 ± 1.1^B,b^	−37.0 ± 1.5^B,b^		7 d	270.4 ± 3.3^B,b^	269.6 ± 1.6^B,b^

*Different letters (A–C) in the same column indicate significant differences between incubation times (p < 0.05). Different letters (a,b) in the same row indicate significant differences between NP concentrations (p < 0.05). Zeta potential and hydrodynamic radius of NPs in DW were measured in the absence of PBS as a control*.

**Figure 7:**
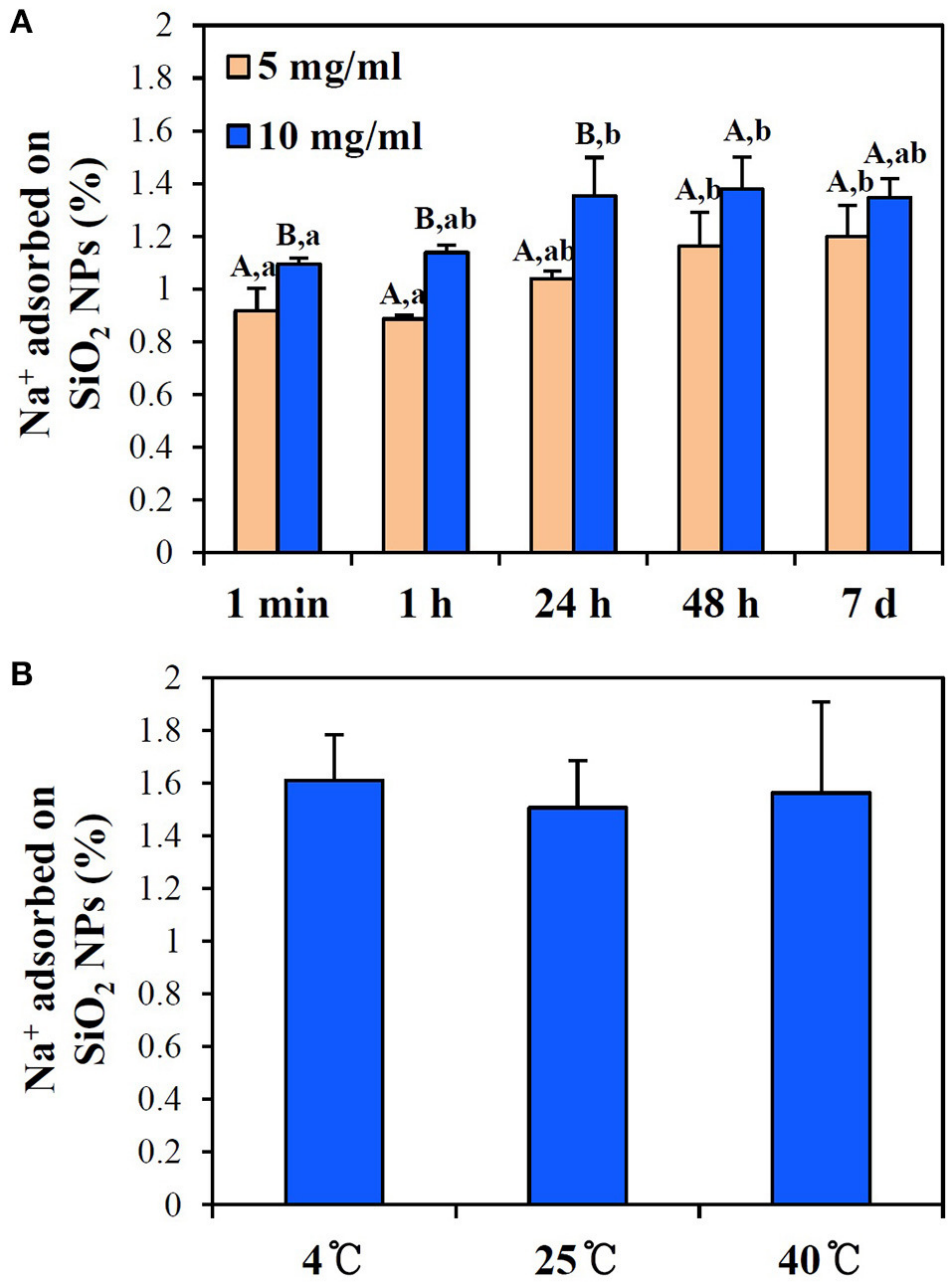
ICP-AES analysis of the interactions between SiO_2_ NPs and Na^+^ in PBS buffer with respect to **(A)** incubation times at 25°C and **(B)** different temperatures at 10 mg/ml NPs after 24 h. **(A)** Different letters in majuscule (A,B) and in minuscule (a,b) indicate significant differences between NP concentrations and between incubation times, respectively (*p* < 0.05). **(B)** No significant differences between different temperatures were found (*p* > 0.05).

Quantitative analysis was also performed by measuring the amount of bound Na^+^ to NPs, the most abundant mineral in PBS, by ICP-AES. As shown in Figure 7A, the interactions between SiO_2_ NPs and Na^+^ increased as NP concentration and incubation time increased. However, no effect of temperature on the interactions was found (Figure 7B; *p* > 0.05). Overall, < 1.7% of Na^+^ was determine to interact with SiO_2_ NPs (Figure 7B).

## Discussion

In the present study, acacia honey, skim milk, olive oil, and PBS buffer were used as representative foods to investigate the interactions between food additive SiO_2_ NPs and food matrices, and then, NP interactions with main components in each representative food were further explored quantitatively. Physicochemical characterization results demonstrate that the primary particle size (24.1 ± 3.5 nm) of SiO_2_ NPs increased in DW, as evidenced by increased hydrodynamic radius (287.6 ± 1.7 nm; Figure 1), indicating their high agglomeration or aggregation tendency. Solubility of SiO_2_ NPs was not detected under all experimental conditions including DW and food matrices, suggesting their particulate fate in food matrices. This result is highly consistent with our previous report (Kim et al., 2016).

SiO_2_ NPs in honey had less negative surface charges and increased hydrodynamic radii as honey concentration increased compared to NPs in DW (Table 1), suggesting NP interactions with honey. Positively charged minor components in honey, such as amino acids and minerals (Cotte et al., 2003, 2004; Conti et al., 2007), seem to play a role in zeta potential changes. When the interactions were further quantified by HPLC analysis, fructose and glucose were found to be adsorbed on the surface of NPs in a honey concentration- and temperature-dependent manner (Figure 2), while no disaccharide, such as sucrose and maltose, did interact with NPs. This result seems to be closely related to the composition of saccharide in acacia honey (fructose: ~42%, glucose: ~30%, sucrose: ~ 0.2%, maltose: ~0.1%). Hence, elevated levels of fructose and glucose in honey contribute to their active interaction with NPs. Indeed, acacia honey contains high sugar concentration composed of various saccharides as well as small amount of amino acids and minerals (Cotte et al., 2004; Conti et al., 2007), and thus, the interactions between SiO_2_ NPs and saccharides are surely influenced by trace nutrients. This hypothesis was assumed by investigating the interactions between NPs and sugar mixtures containing equivalent amounts (1, 2, and 5%) of fructose, glucose, sucrose, and maltose, respectively. Interestingly, zeta potential values of NPs did not statistically change in sugar mixtures as incubation time increased, but slightly decreased values were found at 5% compared to those at 1 or 2% (Table 3). Meanwhile, their hydrodynamic radii decreased as incubation time increased (Table 3), contrary to the results obtained in honey (Table 1). This result supports the role of trace nutrients in the interactions between NPs and saccharides in honey. Moreover, reduced hydrodynamic radii of SiO_2_ NPs in sugar mixtures imply that saccharides can be used as dispersing agents, as reported by other researches (Montero et al., 2012; Maldiney et al., 2014; Strobl et al., 2014).

On the other hand, saccharide concentration- and temperature-dependent interactions between NPs and each saccharide in sugar mixtures were found, regardless of incubation time (Figure 3). However, statistical differences in the interactions between saccharide types were not remarkably observed, suggesting that all saccharide types can be adsorbed on SiO_2_ NPs in a similar manner. Overall interaction amounts of saccharides in honey and sugar mixtures were < 4.6 and 3.1%, respectively, suggesting that a small portion of saccharides can interact with SiO_2_ NPs. It is worth noting that the maximum concentration of honey solution tested was 10%, which contains about 4.2% fructose, 3.0% glucose, 0.02% sucrose, and 0.01% maltose. Whereas, the highest sugar mixture was composed of 5% of each saccharide. It is, therefore, clear that NP interactions with saccharides are facilitated in the presence of small amount of trace nutrients, as indicated by high NP interactions in honey solution (Figure 2) compared to sugar mixtures (Figure 3). In both honey and sugar mixture solutions, incubation time was not a critical factor affecting interactions.

It is interesting to note that incubation of SiO_2_ NPs with more than 2% of honey for 7 d induced strong aggregation, which was less evident in sugar mixtures (Supplementary Figure 1). This is also evidenced by increased hydrodynamic sizes in honey solution (Table 1), contrary to decreased DLS values in sugar mixtures (Table 3). This result also indicates that trace nutrients in honey play a role in the interactions between SiO_2_ NPs or between SiO_2_ NPs and saccharides. Reduced NP interaction with fructose in honey at 7 d post-incubation may be resulted from NP aggregation (Figure 2B, Supplementary Figure 1). On the other hand, no adsorbed saccharides on NPs were detected after washing with DDW, suggesting week interaction force between two materials. It is also probable that high solubility of saccharides in DW also facilitates to detach adsorbed saccharides from NPs during washing process (Alves et al., 2007).

Interactions between SiO_2_ NPs and skim milk or casein were evidenced by changes in zeta potential in a time-dependent manner (Table 4). Interestingly, zeta potentials of NPs in skim milk solution changed to less negative direction, contrary to more negative charges found in casein solution. Skim milk powder is composed of ~34–37% protein (80% casein and 20% whey protein), 50–52% lactose, 8% minerals, and small amount of lipids as well as amino acids (Lagrange, 2005). The difference in composition between skim milk and casein is strongly likely to differently affect zeta potential change. Moreover, the isoelectric point of casein is 4.6, inducing negative charge under physiological condition. This can also induce zeta potential changes of NPs to more negative direction in casein solution. Interestingly, hydrodynamic size of SiO_2_ NPs in both skim milk and casein solutions did not remarkably increase, in particular, as incubation time increased (Table 4). It is known that proteins can play a role as dispersants for NPs (Bihari et al., 2008; Ji et al., 2010; Jo et al., 2016; Vranic et al., 2017), which may explain this result.

Fluorescence quenching ratios of NPs in 1 mg/ml of skim milk solution (Figure 4) were similar to those in 0.35 mg/ml of casein solution (Figure 5), implying that other nutrients in skim milk did not affect NP interaction with proteins. It is notable that aggregation was observed at 25°C after incubation for 7 d (Supplementary Figure 1), probably leading to high fluorescence quenching ratio (Figures 4B, 5B). Less fluorescence quenching at 40°C (Figures 4C, 5C) than 25°C (Figures 4B, 5B) after 7 d might be attributed to protein denaturation, as observed by remarkably high protein precipitation (Supplementary Figure 1). Meanwhile, blue shift indicating protein denaturation or deformation was only observed in casein solution at 5 mg/ml NP concentration at 40°C after 48 h. Moreover, overall fluorescence quenching ratios were < 40%, except incubation for 7 d at 25°C. These results suggest that the interactions between SiO_2_ NPs and proteins are not strong compared to other NP interactions which induce more than 80% quenching (Iosin et al., 2009; Chatterjee et al., 2010; Lee et al., 2015).

On the other hand, small amounts of lipids and minerals were found to interact with SiO_2_ NPs, as evaluated with olive oil and PBS. Oleic acid, palmitic acid, and linoleic acid, main components of olive oil, interacted with NPs without incubation time and temperature effects (Figure 6). However, total adsorbed amounts of oleic acid and palmitic/linoleic acids on NPs were < 1.8 and 0.4%, respectively, indicating that a small portion of fatty acids is adsorbed on NPs. High NP interaction with oleic acid was quantitatively determined because oleic acid is the most abundant fatty acid (~59%) in olive oil. This result is not surprising because SiO_2_ NPs have hydrophilic surface characteristics (Jesionowski and Krysztafkiewicz, 2002), and thus, repulsion force between SiO_2_ NP surfaces and fatty acids is surely predominant. Considering the fact that adsorbed fatty acids on NPs were quantified after subtracting reduced amounts in the supernatant from those in olive oil control, actual adsorbed fatty acids on NP surface seem to be less than the present result obtained. However, the interactions between NPs and lipids could strongly occur for polymer-based NPs or NPs with more hydrophobic surface, such as carbon-based materials. Meanwhile, PBS buffer was used to investigate NP interactions with minerals because mineral composition of PBS is well-known. When the interactions between SiO_2_ NPs and minerals were evaluated by measuring the most abundant mineral component, Na^+^ in PBS, the interactions were dependent on NP concentration and incubation time, but total adsorbed amount was < 1.7% (Figure 7). It should be noted that the interactions were quantified after washing three times with DDW. Hence, it is clear that minerals could be strongly adsorbed on SiO_2_ NPs, although the interaction amount was not that much. This interaction was also supported by significant changes in zeta potential values of NPs to more negative direction and decreased hydrodynamic size in PBS (Table 5). It is strongly likely that small amount of minerals can also act as dispersing agents, by reducing repulsion force between NPs (Dishon et al., 2009).

## Conclusion

In conclusion, food additive SiO_2_ NPs were found to interact with saccharides, proteins, fatty acids, and minerals. Maximum 4.6% of saccharides, 1.7% of fatty acids, and 1.6% of minerals were found to be adsorbed on NPs. The interaction between SiO_2_ NPs and proteins was determined to be rather weak, which did not induce high fluorescence quenching ratio and protein deformation. Trace nutrients were found to play a role in the interactions, as observed by the increased interactions in honey compared to simple sugar mixtures. On the other hand, the degree of NP interaction with skim milk was not much different from that with casein solutions. Saccharides, proteins, and minerals are likely to act as NP dispersants as well. Taken together, SiO_2_ NPs interact with a small portion of food matrices and the interactions are not strong. Further study is required to assume the effects of interactions between NPs and food components on the potential toxicity of NPs as well as the absorption of nutrients and NPs.

## Author contributions

Conceived and designed the experiments and wrote the manuscript: SC. Performed the experiments: MG, SB, HK, and JY. Performed statistical and data analysis: MG and JY.

### Conflict of interest statement

The authors declare that the research was conducted in the absence of any commercial or financial relationships that could be construed as a potential conflict of interest.

## References

[B1] Alves L. A. Silva J. B. A. Giulietti M. (2007). Solubility of D-glucose in water and ethanol/water mixtures. J. Chem. Eng. Data 52, 2166–2170. 10.1021/je700177n

[B2] Bihari P. Vippola M. Schultes S. Praetner M. Khandoga A. G. Reichel C. A.. (2008). Optimized dispersion of nanoparticles for biological *in vitro* and *in vivo* studies. Part. Fibre Toxicol. 5:14. 10.1186/1743-8977-5-14 18990217 PMC2584664

[B3] Bohmert L. Niemann B. Lichtenstein D. Juling S. Lampen A. (2015). Molecular mechanism of silver nanoparticles in human intestinal cells. Nanotoxicology 9, 852–860. 10.3109/17435390.2014.980760 25997095

[B4] Borak B. Biernat P. Prescha A. Baszczuk A. Pluta J. (2012). *In vivo* study on the biodistribution of silica particles in the bodies of rats. Adv. Clin. Exp. Med. 21, 13–18. Available online at: http://www.advances.umed.wroc.pl/en/article/2012/21/1/13/23214294

[B5] Cao Y. Li J. Liu F. Li X. Jiang Q. Cheng S.. (2016). Consideration of interaction between nanoparticles and food components for the safety assessment of nanoparticles following oral exposure: a review. Environ. Toxicol. Pharmacol. 46, 206–210. 10.1016/j.etap.2016.07.023 27497726

[B6] Chatterjee T. Chakraborti S. Joshi P. Singh S. Gupta V. Chakrabarti P. (2010). The effect of zinc oxide nanoparticles on the structure of the periplasmic domain of the *Vibrio cholerae* ToxR protein. FEBS J. 277, 4184–4194. 10.1111/j.1742-4658.2010.07807.x 20825484

[B7] Conti M. E. Stripeikis J. Campanella L. Cucina D. Tudino M. B. (2007). Characterization of Italian honeys (Marche Region) on the basis of their mineral content and some typical quality parameters. Chem. Cent. J. 1:14. 10.1186/1752-153X-1-14 17880749 PMC1994059

[B8] Cotte J. F. Casabianca H. Giroud B. Albert M. Grenier-Loustalot M. F. (2004). Characterization of honey amino acid profiles using high-pressure liquid chromatography to control authenticity. Anal. Bioanal. Chem. 378, 1342–1350. 10.1007/s00216-003-2430-z 14740139

[B9] Cotte J. F. Casabianca H. Chardon S. Lheritier J. Grenier-Loustalot M. F. (2003). Application of carbohydrate analysis to verify honey authenticity. J. Chromatogr. A. 1021, 145–155. 10.1016/j.chroma.2003.09.005 14735983

[B10] Dekkers S. Krystek P. Peters R. J. B. Lankveld D. P. K. Bokkers B. G. H. Hoeven-Arentzen P. H.. (2011). Presence and risks of nanosilica in food products. Nanotoxicology 5, 393–405. 10.3109/17435390.2010.519836 20868236

[B11] Dishon M. Zohar O. Sivan U. (2009). From repulsion to attraction and back to repulsion: the effect of NaCl, KCl, and CsCl on the force between silica surfaces in aqueous solution. Langmuir 25, 2831–2836. 10.1021/la803022b 19437699

[B12] Docter D. Westmeier D. Markiewicz M. Stolte S. Knauer S. K. Stauber R. H. (2015). The nanoparticle biomolecule corona: lessons learned - challenge accepted? Chem. Soc. Rev. 44, 6094–6121. 10.1039/C5CS00217F 26065524

[B13] Dorier M. Brun E. Veronesi G. Barreau F. Pernet-Gallay K. Desvergne C.. (2015). Impact of anatase and rutile titanium dioxide nanoparticles on uptake carriers and efflux pumps in Caco-2 gut epithelial cells. Nanoscale 7, 7352–7360. 10.1039/C5NR00505A 25825056

[B14] Iosin M. Toderas F. Baldeck P. L. Astilean S. (2009). Study of protein-gold nanoparticle conjugates by fluorescence and surface-enhanced Raman scattering. J. Mol. Struct. 924–926, 196–200. 10.1016/j.molstruc.2009.02.004

[B15] Jesionowski T. Krysztafkiewicz A. (2002). Preparation of the hydrophilic/hydrophobic silica particles. Colloid. Surf. A. Eng. Asp. 207, 49–58. 10.1016/S0927-7757(02)00137-1

[B16] Ji Z. Jin X. George S. Xia T. Meng H. Wang X.. (2010). Dispersion and stability optimization of TiO_2_ nanoparticles in cell culture media. Sci. Technol. 44, 7309–7314. 10.1021/es100417s 20536146 PMC3971839

[B17] Jiang Q. Li X. Cheng S. Gu Y. Chen G. Shen Y.. (2016). Combined effects of low levels of palmitate on toxicity of ZnO nanoparticles to THP-1 macrophages. Environ. Toxicol. Pharmacol. 48, 103–109. 10.1016/j.etap.2016.10.014 27770658

[B18] Jo M. R. Chung H. E. Kim H. J. Bae S. H. Go M. R. Yu J. (2016). Effects of zinc oxide nanoparticle dispersants on cytotoxicity and cellular uptake. Mol. Cell. Toxicol. 12, 281–288. 10.1007/s13273-016-0033-y

[B19] Kim M. K. Lee J. A. Jo M. R. Choi S. J. (2016). Bioavailability of silica, titanium dioxide, and zinc oxide nanoparticles in rats. J. Nanosci. Nanotechnol. 16, 6580–6586. 10.1166/jnn.2016.12350 27427756

[B20] Lagrange V. (2005). Reference Manual for US and Milk Powders 2005 Revised Edn. Arlington, VA: US Dairy Export Council.

[B21] Lee J. A. Kim M. K. Kim H. M. Lee J. K. Jeong J. Kim Y. R.. (2015). The fate of calcium carbonate nanoparticles administered by oral route: absorption and their interaction with biological matrices. Int. J. Nanomed. 10, 2273–2293. 10.2147/IJN.S79403 25848250 PMC4376267

[B22] Lee J. A. Kim M. K. Paek H. J. Kim Y. R. Lee J. K. Jeong J. (2014). Tissue distribution and excretion kinetics of orally administered silica nanoparticles in rats. *Int*. J. Nanomed. 9, 251–260. 10.2147/IJN.S57939 PMC4279759 25565843

[B23] Lee J. A. Kim M. K. Song J. H. Jo M. R. Yu J. Kim K. M.. (2017). Biokinetics of food additive silica nanoparticles and their interactions with food components. Colloid. Surf. B. 150, 384–392. 10.1016/j.colsurfb.2016.11.001 27842933

[B24] Liang N. N. Zhang L. X. Wang X. L. Tan B. B. Liang Y. Z. (2011). Identification of fatty acids in vegetable oils by mass spectrometry and equivalent chain length. Chin. J. Anal. Chem. 39, 1166–1170. 10.3724/SP.J.1096.2011.01166

[B25] Lichtenstein D. Ebmeyer J. Knappe P. Juling S. Böhmert L. Selve S.. (2015). Impact of food components during *in vitro* digestion of silver nanoparticles on cellular uptake and cytotoxicity in intestinal cells. Biol. Chem. 396, 1255–1264. 10.1515/hsz-2015-0145 26040006

[B26] Mahler G. J. Esch M. B. Tako E. Southard T. L. Archer S. D. Glahn R. P.. (2012). Oral exposure to polystyrene nanoparticles affects iron absorption. Nat. Nanotechnol. 7, 264–271. 10.1038/nnano.2012.3 22327877

[B27] Maldiney T. Bessière A. Seguin J. Teston E. Sharma S. K. Viana B.. (2014). The *in vivo* activation of persistent nanophosphors for optical imaging of vascularization, tumours and grafted cells. Nat. Mater. 13, 418–426. 10.1038/nmat3908 24651431

[B28] Montero M. Molina T. Szafran M. Moreno R. Nieto M. I. (2012). Alumina porous nanomaterials obtained by colloidal processing using D-fructose as dispersant and porosity promoter. Ceram. Int. 38, 2779–2784. 10.1016/j.ceramint.2011.11.048

[B29] Paek H. J. Chung H. E. Lee J. A. Kim M. K. Lee Y. J. Kim M. S. (2014). Quantitative determination of silica nanoparticles in biological matrices and their pharmacokinetics and toxicokinetics in rats. Sci. Adv. Mater. 6, 1605–1610. 10.1166/sam.2014.1817

[B30] Strobl F. G. Seitz F. Westerhausen C. Reller A. Torrano A. A. Bräuchle C.. (2014). Intake of silica nanoparticles by giant lipid vesicles: influence of particle size and thermodynamic membrane state. Beilstein J. Nanotechnol. 5, 2468–2478. 10.3762/bjnano.5.256 25671142 PMC4311713

[B31] Vance M. E. Kuiken T. Vejerano E. P. McGinnis S. P. Hochella M. F. Jr. Rejeski D.. (2015). Nanotechnology in the real world: redeveloping the nanomaterial consumer products inventory. Beilstein J. Nanotechnol. 6, 1769–1780. 10.3762/bjnano.6.181 26425429 PMC4578396

[B32] Vranic S. Gosens I. Jacobsen N. R. Jensen K. A. Bokkers B. Kermanizadeh A.. (2017). Impact of serum as a dispersion agent for *in vitro* and *in vivo* toxicological assessments of TiO_2_ nanoparticles. Arch. Toxicol. 91, 353–363. 10.1007/s00204-016-1673-3 26872950

[B33] Wang H. Du L. J. Song Z. M. Chen X. X. (2013). Progress in the characterization and safety evaluation of engineered inorganic nanomaterials in food. Nanomedicine 8, 2007–2025. 10.2217/nnm.13.176 24279490

[B34] Wang Y. Yuan L. Yao C. Ding L. Li C. Fang J.. (2014). A combined toxicity study of zinc oxide nanoparticles and vitamin C in food additives. Nanoscale 6, 15333–15342. 10.1039/C4NR05480F 25387158

