# Supplementary material for: Interactions between Food Additive Silica Nanoparticles and Food Matrices
Source: Front Microbiol. 2017 Jun 7;8:1013. doi: 10.3389/fmicb.2017.01013 (PMC5461366; doi:10.3389/fmicb.2017.01013)
Supplement: Supplementary file 1 [file Presentation1.PDF]

Supplementary Material

Interactions between food additive silica nanoparticles and food matrices

Mi-Ran Go, Song-Hwa Bae, Hyeon-Jin Kim, Jin Yu, and Soo-Jin Choi*

*** Correspondence:** Soo-Jin Choi: sjchoi@swu.ac.kr

1.1 Supplementary Figure

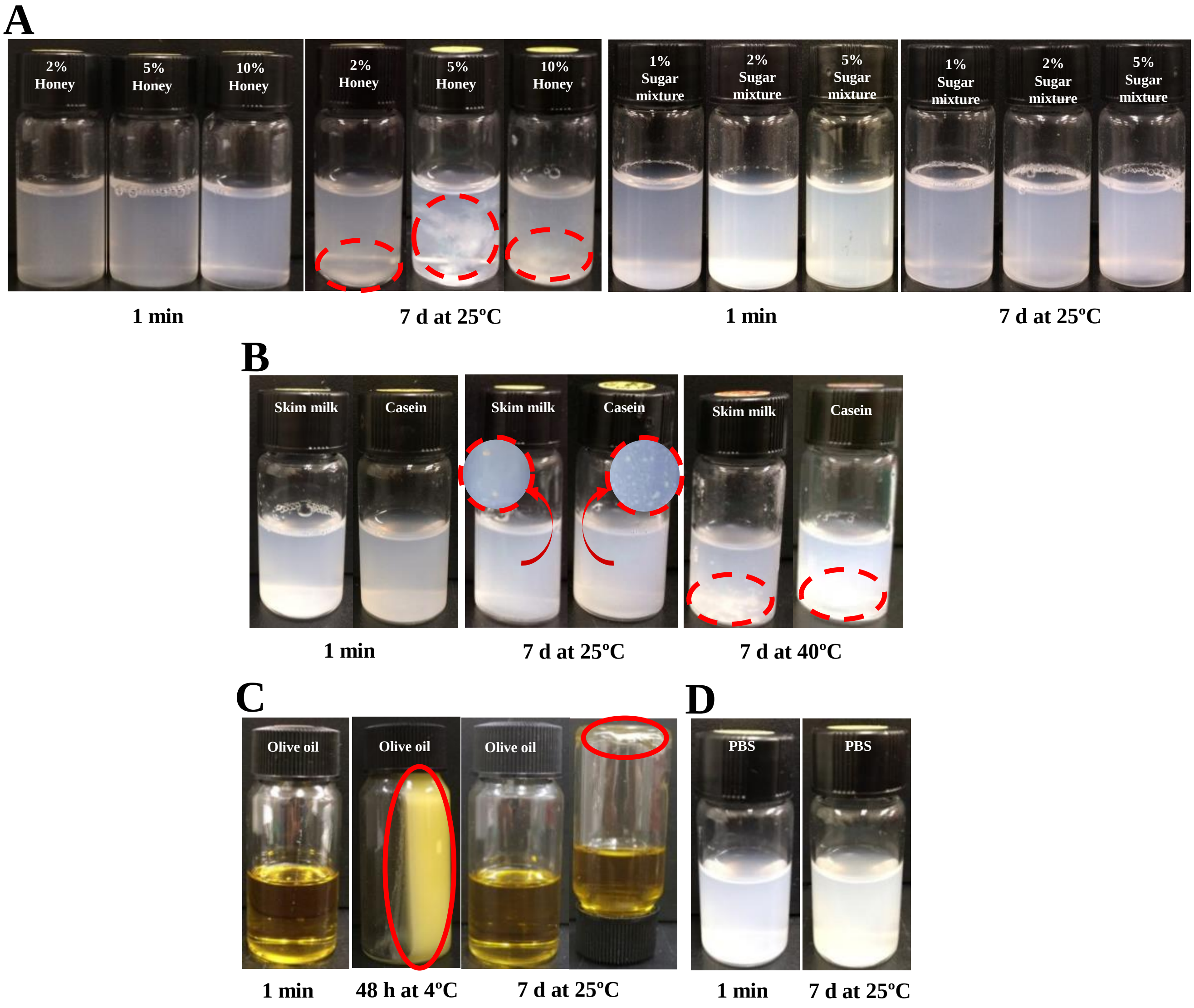

Supplementary Figure 1. Photos of SiO_2 NPs in (A) different concentrations of honey or sugar mixture, (B) skim milk solution (1 mg/ml) or casein solution (0.35 mg/ml), (C) olive oil, and (D) PBS buffer, showing aggregation or gelation.
