# Supplementary material for: Interactions between Food Additive Silica Nanoparticles and Food Matrices
Source: Front Microbiol. 2017 Jun 7;8:1013. doi: 10.3389/fmicb.2017.01013 (PMC5461366; doi:10.3389/fmicb.2017.01013)
Supplement: Supplementary file 2 [file Presentation2.PDF]

1.2 Supplementary Figure

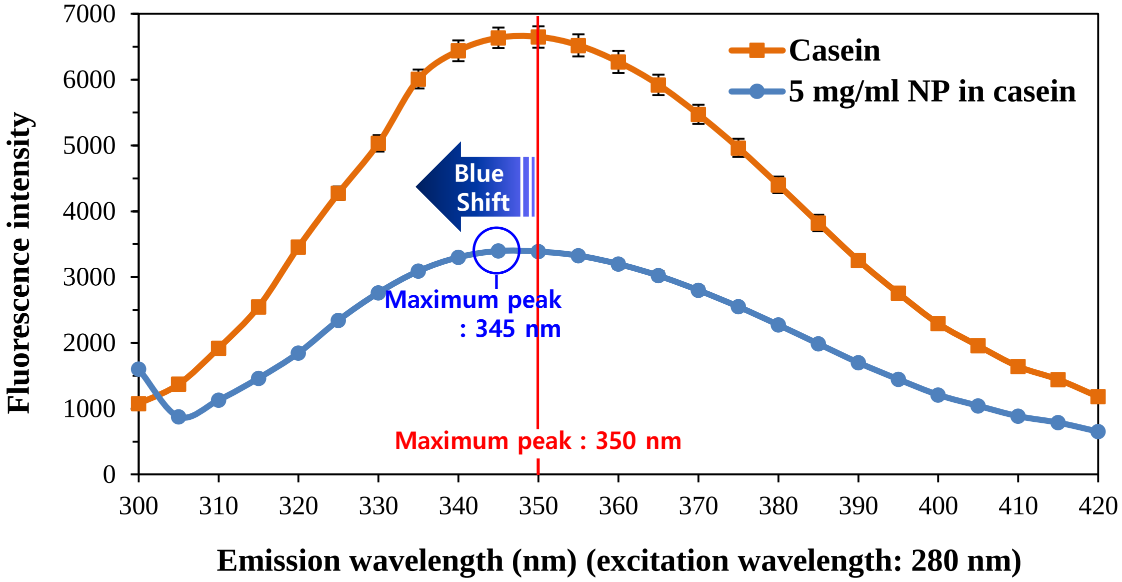

Supplementary Figure 2. Fluorescence spectra of casein in the absence or in the presence of 5 mg/ml SiO_2 NPs at 40ºC after 48 h.
